# Analytical validation of Exandra: a clinical decision support system for promoting guideline-directed therapy of type-2 diabetes in primary care – a collaborative study with experts from Diabetes Canada

**DOI:** 10.1186/s12911-025-02881-4

**Published:** 2025-02-12

**Authors:** Klaudia Grechuta, Pedram Shokouh, Valentina Bayer, Henrich Kraemer, Jeremy Gilbert, Susie Jin, Ahmad Alhussein

**Affiliations:** 1 Boehringer Ingelheim International GmbH, Binger Straße 173, Ingelheim am Rhein, 55216 Germany; 2 Adivus Medical Consultancy Mpv., Aarhus, Denmark; 3 Boehringer Ingelheim Pharmaceuticals, Inc., Ridgefield, CT USA; 4 Sunnybrook Health Sciences Centre, University of Toronto, Toronto, Canada; 5 Clinical Pharmacist, Certified Diabetes Educator, Cobourg, Ontario Canada

**Keywords:** Type 2 diabetes mellitus, Clinical decision support systems, Comorbidity

## Abstract

**Background:**

Individuals with type 2 diabetes (T2D) have a high prevalence of cardiovascular and renal comorbidities. Despite clinical practice guidelines recommending the use of cardiorenal protective medications, many people with T2D are not prescribed these medications. A clinical decision support system called Exandra was developed to provide treatment recommendations for individuals with T2D based on current clinical practice guidelines from Diabetes Canada. The current study aimed to medically validate Exandra via review by external medical experts in T2D.

**Methods:**

Validation of Exandra took place in two phases. Test cases using simulated clinical scenarios and recommendations were generated by Exandra. In Phase 1 of the validation, reviewers evaluated whether they agreed with Exandra’s recommendations with a “yes,” “no,” or “not sure” response. In Phase 2, reviewers were interviewed about their “no” and “not sure” responses to determine possible reasons and potential fixes to the Exandra system. The primary outcome was the precision rate of Exandra following the interviews and final adjudication of the cases. The target precision rate was 90%.

**Results:**

Exandra displayed an overall precision rate of 95.5%. A large proportion of cases that were initially labeled “no” or “not sure” by reviewers were changed to “yes” following the interview phase. This was largely due to the validation using a simplified user interface compared with the complexity of the actual Exandra system, and reviewers needing clarification of how the outputs would be displayed on the Exandra platform.

**Conclusion:**

Exandra displayed a high level of accuracy and precision in providing guideline-directed recommendations for managing T2D and its common comorbidities. The results of this study indicate that Exandra is a promising tool for improving the management of T2D and its comorbidities.

**Supplementary Information:**

The online version contains supplementary material available at 10.1186/s12911-025-02881-4.

## Background

Individuals with type 2 diabetes (T2D) have a high prevalence of cardiovascular and renal comorbidities [1, 2]. Recent guidelines for the treatment of T2D now include the use of cardiorenal protective medications such as sodium-glucose cotransporter-2 inhibitors (SGLT2is) and glucagon-like peptide-1 receptor agonists (GLP-1RAs) [3–5]. SGLT2is are oral anti-hyperglycemic medications that have also been shown to have weight loss, antihypertensive, cardiovascular, and cardiorenal benefits [4, 6, 7]. GLP-1RAs have similarly been shown to reduce the rate of major adverse cardiovascular events, in addition to reducing weight, blood pressure, and inflammation [8]. Despite this, most individuals with T2D who are at increased risk of cardiovascular and renal complications do not receive SGLT2is or GLP-1RAs [9]. In one US study, less than 2% of individuals with T2D were treated with either an SGLT2i or GLP-1RA [10]. Most participants in the study were treated with insulin (41%), biguanides (20%), sulfonylurea (9%), and dipeptidyl peptidase-4 (DPP-4) inhibitors (6%). Possible reasons for the lack of adherence to diabetes guidelines as a whole include lack of reimbursement, time constraints, the need for individualized treatment, insufficient human resources, and therapeutic inertia [11–13].

For a time-constrained general practitioner, consolidating numerous guidelines to make clinical decisions is challenging. This is particularly true for cardiovascular disease, where overall or absolute risk assessment is recommended, and simultaneous management of multiple risk factors and complications is required. Surveyed physicians have reported that electronic therapy decision support may help increase guideline-directed care [14]. Clinical Decision Support Systems (CDSSs) are a promising solution that can inform and assist healthcare professionals in keeping up-to-date with the latest clinical practice guidelines [15, 16]. A number of CDSSs have been developed with the goal to improve diabetes and cardiovascular risk management [16–21]. CDSSs have been shown to reduce hospitalizations [17], improve risk management [18, 19], provide better care in low-income settings [20], and improve quality of treatment while reducing cost [21].

Exandra is a standalone, web-based CDSS, designed to assist healthcare professionals in making informed treatment decisions for individuals with T2D. The tool’s algorithms are designed to receive clinical and laboratory parameters and provide personalized therapeutic recommendations based on integrated local guidelines to achieve glycemic targets and cardiorenal protection in non-hospitalized people. Healthcare professionals can independently review the basis for each recommendation and make informed decisions regarding the treatment of the individual. This study aimed to validate Exandra and ensure it was medically accurate prior to launch via expert medical review.

## Methods

### Development of the decision engine

Exandra operates as a knowledge-based CDSS, using a multi-component decision engine that was built through collaboration between medical experts and an engineering team from Boehringer Ingelheim (BI) and its digital lab. Internal medical experts acquired data to build the engine from official reference guideline chapters published by Diabetes Canada, as well as a number of secondary sources (see Supplementary Table S1 for full list of references). Patient parameters were defined using guideline information (see Supplementary Table S2 for full list of patient parameters), and treatment recommendations associated with every combination of parameters (i.e., clinical scenarios) were extracted from guideline texts to form an exhaustive set of decision rules. A second BI expert verified and approved the process at every step.

**Fig. 1:**
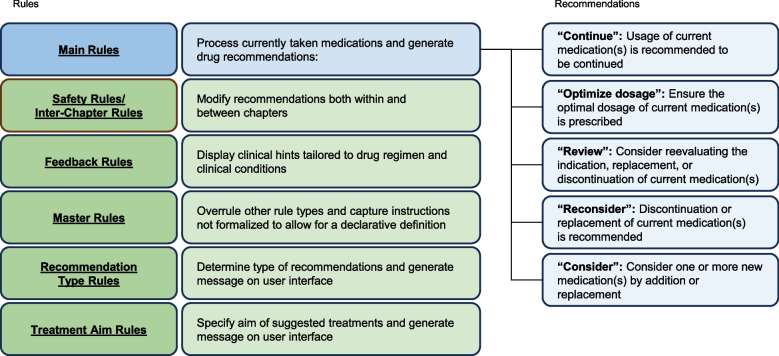
Rules used by Exandra and recommendations generated by Main Rules

The engine was divided into separate blocks called chapters, representing the therapeutic areas covered by Exandra. The chapters were: 1. Glycemic management, 2. Dyslipidemia, 3. Blood pressure control, and 4. Anti-platelet therapy. Each chapter was segmented into different types of rules, detailed in Fig. 1. The Main Rules were classified into clusters that were allocated to a defined group of clinical scenarios. Main Rules were those that processed currently taken medications and generated drug recommendations based on a combination of clinical parameters. Figure 1 details each of the drug recommendations generated by Main Rules. Safety Rules and Inter-Chapter Rules were used to modify recommendations for safety reasons both within and between chapters (e.g., for drug–drug interactions or contraindications). Feedback Rules displayed clinical hints tailored to each patient’s drug regimen and clinical conditions. Master Rules were those that could overrule other rule types and captured instructions not formalized to allow for a declarative definition. Recommendation Type Rules determined the type of recommendations (i.e., “by addition” or “by replacement”) and Treatment Aim Rules specified the aim of the suggested treatments (i.e., “for cardiorenal protection” or “for glycemic control”) by generating a message on the user interface. Exandra also encompassed other types of logic to generate information such as dose recommendations, renal dose adjustments, and verifying the validity of inputs. Exandra only recognized medicinal substances, rather than pharmaceutical products, and was brand agnostic. Medicinal substances were classified into medication classes.

**Fig. 2:**
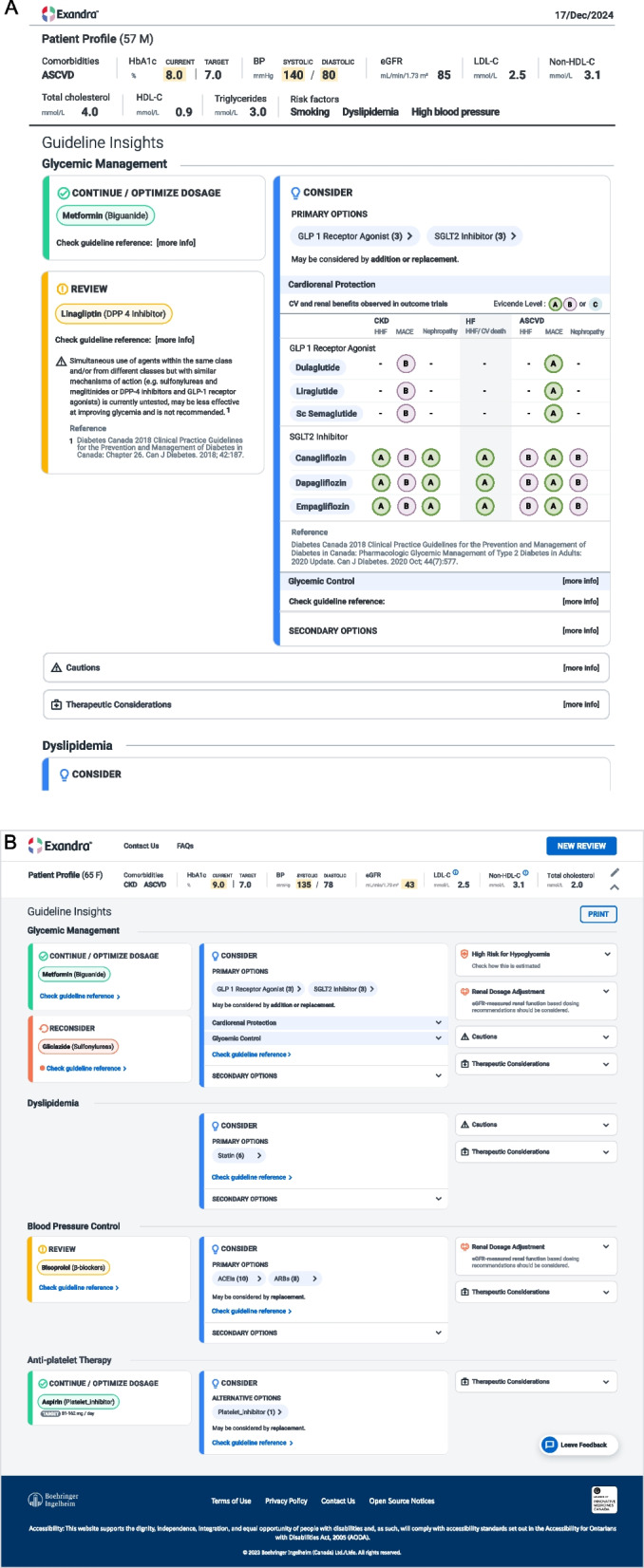
**A** and **B** Screenshots of user interface presenting examples of Exandra recommendation outputs. ASCVD, atherosclerotic cardiovascular disease; BP, blood pressure; CKD, chronic kidney disease; CV, cardiovascular; DPP-4, dipeptidyl peptidase 4; eGFR, estimated glomerular filtration rate; GLP-1, glucagon-like peptide-1; HbA1c, glycated hemoglobin; HDL-C, high-density lipoprotein cholesterol; HF, heart failure; HHF, hypertensive heart failure; LDL-C, low-density lipoprotein cholesterol; MACE, major adverse cardiovascular event; M, male; non-HDL-C, non-high-density lipoprotein cholesterol; SGLT2, sodium-glucose transport protein 2

Figures 2A and B present the user interface of Exandra and example patient cases. The recommendations generated by the decision engine were based on official references published by Canadian professional nonprofit organizations such as Diabetes Canada. For each recommendation, the engine provided a complete list of references to enable independent appraisal of the content (Fig. 2A). The Diabetes Canada Clinical Practice Guidelines were regarded as the primary source, and if required, secondary sources and product monographs available in Canada were consulted and cited as supporting evidence. On rare occasions, the Diabetes Canada Clinical Practice Guidelines would not provide a univocal treatment recommendation. Gray areas in the body of knowledge were bridged by expert opinion from collaborating specialists appointed by Diabetes Canada. See Supplemental Methods for a list of references used for the development of Exandra per chapter.

### Technical validation

For technical validation of the engine, the engineering team collaborated closely with the medical experts so that the algorithmic descriptions authored by the experts could seamlessly be converted into a computable format, and the engineers could provide testing tools to allow medical experts to verify them. Established software engineering practices were followed to produce quality code, including employing a quick feedback cycle, running automated tests on every build, peer review of all code changes, and a continuous integration pipeline. To verify that the engine performed as expected, the engineers completed a set of tests confirming that basic types and mechanisms for the engine worked. Under supervision of the medical experts, the engine framework was designed and implemented to generate random test cases.

### Test case generation

**Fig. 3:**
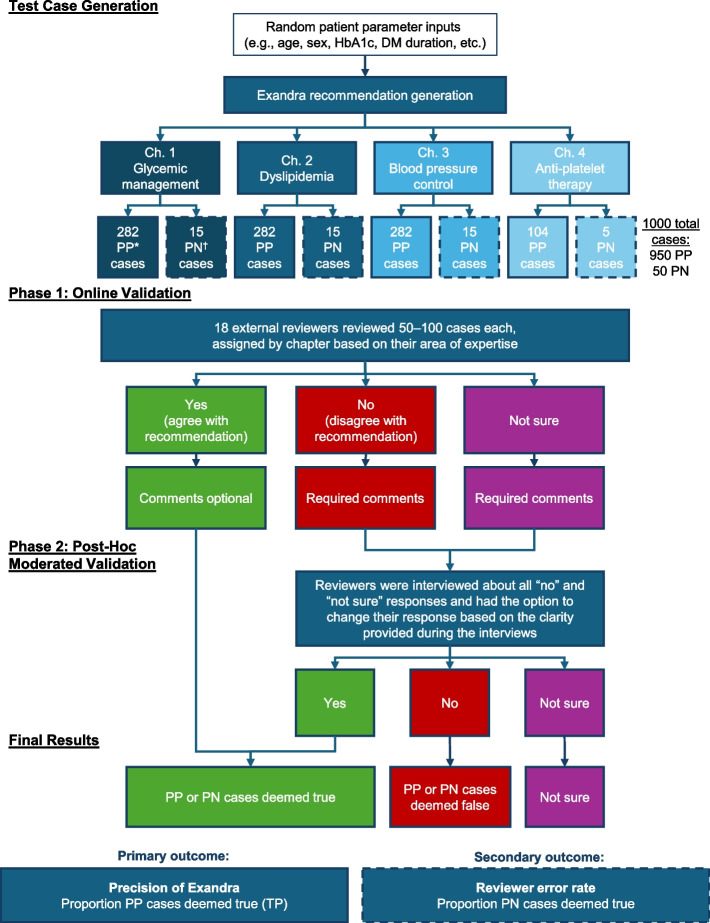
Methodology of Exandra validation. *PP cases are those that have been generated by Exandra for external expert validation. ^†^PN cases are generated test cases that have been manually converted into false cases by swapping and altering the treatment recommendations generated by Exandra to ensure the contents did not conform with guideline recommendations. DM, diabetes mellitus; HbA1c, glycated hemoglobin; PN, predicted negative; PP, predicted positive; TP, true positive

Exandra was validated to verify its accuracy via review by medical experts from the development team, as well as independent experts appointed by Diabetes Canada. A summary of the methodology is provided in Fig. 3. To do this, each chapter of the decision engine was validated through simulated clinical scenarios and their Exandra-generated recommendations, referred to as test cases. A test case consisted of: 1. A random combination of chapter-specific patient clinical parameters generated by a random case generator, and 2. Recommendations generated by the Exandra decision engine (also called “prediction labels”).

To generate medically plausible test cases, constraints were defined for the values that clinical parameters could assume and possible numbers and variations of medications. A random case generator followed the constraints and ensured statistical randomness was achieved. The required number of random cases needed to achieve target confidence interval (CI) was determined, and this number of case inputs was generated by the random case generator. To form complete test cases, recommendations were generated by Exandra for each random input, and both random case inputs and their respective recommendations were recorded as full test cases. For each chapter, a baseline test suite was created, and automated test suites were run for each build of Exandra and compared against the baseline recommendations to verify that the case recommendations did not change unless desired and medically reviewed.

### Internal expert validation

The first step toward validating the accuracy of Exandra was the internal validation phase. Two medical experts independently reviewed a set of 950 random test cases for medical accuracy. The experts cross-referenced all components of the test cases with cited references to determine whether generated outputs were medically correct and aligned with their expectations. The target precision level for this phase of review was 100% in all chapters (i.e., no disagreement with the references/guidelines). Any level of inaccuracy was marked and documented, and for each issue, a fix was devised, and the engine was amended. After each engine fix, the same test cases were generated and reviewed, and this reiterative process was continued until 100% accuracy was reached.

### External expert validation

In the second step toward validation, independent reviewers from Diabetes Canada reviewed test cases and assessed whether the treatment recommendations generated by Exandra’s engine were in line with the guidelines. For this evaluation, a new set of random test cases was generated, called predicted positive (PP) cases. To estimate the rate of reviewer error and control for subjectivity or bias, 5% of the generated test cases were manually converted into false cases (referred to as predicted negative [PN] cases) by swapping and altering the treatment recommendations generated by Exandra to ensure the contents did not conform with guideline recommendations. These PN cases were equally and randomly distributed along with the PP cases presented to the reviewers. The label of PN or PP was visible to the experimenters only, and not to the external reviewers.

**Fig. 4:**
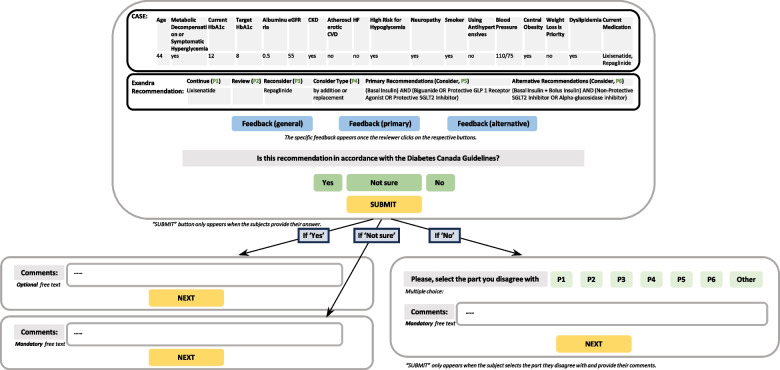
Example of the evaluation form answer flow diagram. P1-P6 refer to different parts of the recommendation. CKD, chronic kidney disease; CVD, cardiovascular disease; eGFR, estimated glomerular filtration rate; GLP 1, glucagon-like peptide 1; HbA1c, glycated hemoglobin; HF, heart failure; SGLT2, sodium-glucose transporter 2

External validation took place in two phases: 1. Online validation performed by the reviewers, and 2. Post-hoc moderated validation. In Phase 1, cases were presented to the reviewers in a user-friendly format accessible online. Each test case was presented consecutively so the reviewer had to submit an answer prior to viewing the next case. All reviewers received standardized instructions on how to complete the assessment in a face-to-face onboarding session. Reviewers were instructed to respond to each test case with “yes”, “no”, or “not sure”. A “yes” answer indicated the reviewer assessed the recommendation as consistent with the Diabetes Canada guidelines. A “no” answer meant significant or complete disagreement of the reviewer with the recommendations generated by Exandra. A “not sure” answer was used if the reviewer could not tell whether a recommendation was correct due to insufficient information for adjudication. If a reviewer answered “no” or “not sure” they were required to provide a comment about which parts of the recommendation they disagreed with, and their rationale. Providing notes or clarifications was optional for “yes” answers (Fig. 4). Reviewers were encouraged to use the Diabetes Canada guidelines to support their judgments and comments. All responses were anonymized and securely stored for further quantitative and qualitative analyses.

In Phase 2, reviewers participated in an open-ended interview about their “no” or “not sure” responses to understand the rationale behind the ratings and revise the adjudication if applicable. In order to showcase the functions of the system and minimize the possibility of misunderstanding of the study protocol or how Exandra works, a live demo of Exandra was presented prior to the interviews. Each “no” or “not sure” case was critically discussed, and reviewers were given the option to alter their initial adjudication in any way they saw fit. Responses during the interviews were documented, and post-interview “yes,” “no,” and “not sure” responses were considered the final results of the validation study.

Details of how the required sample size of test cases was calculated are provided in the Supplementary Methods. A total of 1000 random test cases were generated, including 271 test cases from each of the larger chapters (1–3), 101 cases from the smaller chapter (4), and 86 additional “buffer” test cases evenly distributed among the chapters to bring the total to 1000. Fifty cases (5%) were manually converted to PN cases, and the remaining 950 cases were PP. Each reviewer was presented with 50 cases to independently review (with the exception of two reviewers, who were presented with 100 cases each). Each reviewer received cases from one chapter only, based on their area of expertise.

Those PP cases that were accepted by the reviewers as correct were considered the “true positive” (TP) cases (Table 1). The primary outcome was the precision after the final adjudication, defined as the proportion of TP cases out of PP cases: Precision = TP/PP = TP/(TP + false positive [FP]). The target precision level for the external validation stage was 90% or more in all chapters, based on the acceptance rate of other diabetes CDSSs in the literature [22]. The secondary outcome was the proportion of PN cases in which the reviewers agreed with the incorrect treatment recommendation (true negative, TN); this was the reviewer error rate (also called negative predictive value) = TN/PN. The accuracy of Exandra was defined as (TP + TN) / total cases.

**Table 1:** Matrix showing the definition of PP, TP, and FP test cases

	**Actual positive (according to DC after final adjudication)**	**Actual negative (according to DC after final adjudication)**
**PP (used for the calculation of precision)**	TP (the reviewer agreed with Exandra’s prediction of a positive case, “yes”)	FP (the reviewer disagreed with Exandra’s prediction of a positive case, “no”)

*DC* Diabetes Canada, *FP* false positive, *PP* predicted positive, *TP* true positive

## Results

### Generation and assignment of test cases

Of the 914 required test cases, 30% were from each of Chapters 1–3, and 10% were from Chapter 4. The 86 additional “buffer” cases were evenly distributed among the chapters. Five percent of the 1000 total test cases (i.e., 50 cases) were randomly selected and manually converted to PN cases (15 cases in each of Chapters 1–3 and five cases in Chapter 4). There were 18 participating external reviewers, and each received a link to access cases from a single chapter, assigned based on their area of expertise (16 reviewers received 50 cases each, and two reviewers received 100 each). Table 2 presents the numbers of PP and PN test cases generated per chapter. There was a total of 950 PP and 50 PN cases. After the final adjudication, there were four cases in which the reviewers’ labels were missing (all were PP cases from Chapter 1), resulting in a total of 996 cases.

**Table 2:** Test cases generated for external validation study

Study Phase	Total	Chapter 1. Glycemic management	Chapter 2. Dyslipidemia	Chapter 3. Blood pressure control	Chapter 4. Anti-platelet therapy
**Phase 1 (online validation)**					
Random test cases (PP)	950	282	282	282	104
Test cases converted to PN	50	15	15	15	5
Total	1000	297	297	297	109
**Phase 2 (post-adjudication/final)**					
Missing	4	4	0	0	0
Final valid *n*	996	293	297	297	109
Final valid % of all cases	100	29	30	30	11

*PN* predicted negative, *PP* predicted positive

### Phase 1 (online validation)

Prior to the qualitative interview, reviewers labeled the cases with either “yes” to indicate they agreed with Exandra’s recommendation, “no” to indicate they did not agree with the recommendation, or “not sure” if they were unsure about the recommendation. Among the 950 PP cases, a total of 556 were labeled “yes”, 259 were “no”, and 135 were “not sure” (Table 3). Among the 50 PN cases, six were labeled “yes”, 35 were “no”, and nine were “not sure.” Overall, 58.5% of the PP cases were labeled “yes”.

**Table 3:** Reviewer responses during Phase 1 of external validation study

Response	Total (*N* = 1000)	Chapter 1. Glycemic management (*n* = 297)	Chapter 2. Dyslipidemia (*n* = 297)	Chapter 3. Blood pressure control (*n* = 297)	Chapter 4. Anti-platelet therapy (*n* = 109)
**PP**					
Yes	556	151	179	144	82
No	259	92	70	92	5
Not sure	135	39	33	46	17
**PN**					
Yes	6	1	2	1	2
No	35	11	11	11	2
Not sure	9	3	2	3	1

*PN* predicted negative, *PP* predicted positive

Upon qualitative review of the comments associated with the “no” responses, a high ratio of the rejected cases was based on minor discrepancies, which, based on the protocol, were not sufficient grounds for rejection. In several cases, the rejection appeared to have stemmed from a lack of familiarity with the way recommendations were presented in the tool. For example, the reviewer was unclear whether the display of multiple primary treatment options signaled their equal clinical value or not. To verify the grounds for case rejections, qualitative interviews were conducted with the reviewers in Phase 2 to discuss all “no” and “not sure” responses to PP cases and adjudicate the responses (if applicable).

### Phase 2 (qualitative interviews and final adjudication)

Among the 259 PP cases initially labeled “no” by reviewers, 228 were changed to “yes” following the qualitative interviews (Table 4). Of the 135 PP cases labeled “not sure”, 110 were changed to “yes.” Following the qualitative interviews and final adjudication, 10 cases remained labeled “not sure” (Table 5). The “not sure” cases were not included in the calculation for precision.

**Table 4:** Changes in “no” and “not sure” responses to PP cases after Phase 2 (final adjudication)

Response After Phase 2	Total	Chapter 1. Glycemic management (*n* = 124)	Chapter 2. Dyslipidemia (*n* = 111)	Chapter 3. Blood pressure control (*n* = 135)	Chapter 4. Anti-platelet therapy (*n* = 11)
**PP cases marked “no” in Phase 1**	259	92	70	92	5
“No” to “yes”	228	79	66	82	1
“No” to “not sure”	4	0	1	2	1
Unchanged “no”	26	12	3	8	3
Missing	1	1	0	0	0
**PP cases marked “not sure” in Phase 1**	135	39	33	46	17
“Not sure” to “yes”	110	33	31	41	5
“Not sure” to “no”	16	3	2	1	10
Unchanged “not sure”	6	0	0	4	2
Missing	3	3	0	0	0

*PP* predicted positive

**Table 5:** Calculated Exandra precision

Response to PP case	Total (*N* = 946)	Chapter 1. Glycemic management (*n* = 293)	Chapter 2. Dyslipidemia (*n* = 297)	Chapter 3. Blood pressure control (*n* = 297)	Chapter 4. Anti-platelet therapy (*n* = 109)
**Yes (TP)**^a^	894	263	276	267	88
**No (FP)**^b^	42	15	5	9	13
**Not sure**^c^	10	0	1	6	3
**Exandra precision ratio (%)**^d^	95.5	94.6	98.2	96.7	87.1

*FP* false positive, *PP* predicted positive, *TP* true positive

^a^Yes (TP) = 894 because 556 “yes” from Phase 1 (Table 3) + 228 “no” cases changed to “yes” from Phase 2 (Table 4) + 110 “not sure” cases changed to “yes” from Phase 2 (Table 4) = 894

^b^No (FP) = 42 because 26 unchanged “no” cases from Phase 2 (Table 4) + 16 “not sure” cases changed to “no” in Phase 2 (Table 4) = 42

^c^Not sure = 10 because 6 unchanged “not sure” cases from Phase 2 (Table 4) + 4 “no” cases changed to “not sure” in Phase 2 (Table 4) = 10

^d^Precision = TP/PP = TP/(TP + FP). This calculation excludes the “not sure” cases

Overall, the precision of Exandra was 95.5%, ranging from 87.1% in the Anti-platelet therapy chapter (Chapter 4), to 98.2% in the Dyslipidemia chapter (Chapter 2) (Table 5). The precision was 94.6% in the Glycemic management chapter (Chapter 1) and 96.7% in the Blood pressure control chapter (Chapter 3). During the online validation phase (Phase 1), 35 of the 50 PN cases were correctly marked “no”, and six were incorrectly marked “yes” by reviewers (Table 3). Therefore, the reviewer error rate was 6/50, or 12%. The PN cases were not included in the qualitative interview phase (Phase 2).

## Discussion

This validation study demonstrated the feasibility of developing a medically accurate CDSS for the management of T2D and its comorbid conditions. It also provided a robust method for validation of the CDSS. Our findings showed that Exandra generated recommendations with a high level of medical precision and conformity with expert interpretation of reference guidelines.

Reported accuracy of all chapters changed after qualitative interviews were conducted. There was a considerable shift from “no” and “not sure” responses toward acceptance of Exandra’s outputs. This shift could be due to several factors. First, the initial online review was conducted using a simplified user interface, which lacked the complexity of Exandra’s actual environment and prompted occasional misinterpretations. For instance, when both statins and ezetimibe were displayed as primary lipid-lowering options, some reviewers interpreted this to mean both options were equal or interchangeable. However, the design of the tool’s user interface is intended to prevent such misunderstandings, and this issue was effectively addressed by presenting the live demo of the fully implemented CDSS to the reviewers. Additionally, since the test cases were generated on a single chapter basis, each case failed to encompass all areas covered by Exandra. Thus, an abridged clinical picture and therapeutic approach was presented. This limitation caused some reviewers to wonder why certain required treatments (included in other chapters) were absent from the generated recommendations in the test cases. This issue was also largely clarified by demoing the tool and the full spectrum of information offered by Exandra.

In some cases, reviewers relied on personal interpretations, or more up-to-date data to review the cases, rather than the literal meaning of the cited guidelines. In these cases, further clarification and reference to cited guidelines were offered during the interview phase. In some rare occasions, the data included in a test case were not sufficient for determining guideline-directed treatments, even after providing additional clarification during the interview. The adjudication results of such cases remained “not sure.”

Despite documenting a high level of medical precision, the external validation study also unveiled a few remaining fixes needed in the test version of the engine. Fixes included suggestions for modifying recommended medications in certain clinical scenarios. For example, pursuant to discussion with Diabetes Canada experts, it was decided to recognize and reflect the clinical differences in the management of ‘metabolic decompensation’ and ‘symptomatic hyperglycemia’, which were combined in Diabetes Canada Clinical Practice Guideline [5]. As a result, the algorithm was adjusted to recommend insulin, without metformin, as the primary treatment in cases with metabolic decompensation. Suggestions for improvement included introducing additional parameters and altering the placement or design of certain outputs in the user interface. Internal medical experts filed all reported proposals for fixes received from adjudicating experts and turned them into actionable items. The items were classified based on their clinical significance. High priority updates were given precedence and were fixed before the release of Exandra. The remaining items were filed into a pipeline of engine updates to be addressed at the earliest possibility and released as post-launch updates. It is important to note that no major safety issues were reported with the test cases by external reviewers. Exandra focuses on offering treatment suggestions for people with T2D, including those with cardiovascular and renal comorbidities such as heart failure, ASCVD, and/or CKD, emphasizing the preference of cardiorenal-protective medications. Additionally, while Exandra is a CDSS that provides recommendations for patients in the outpatient setting, future CDSSs should be developed that address patients in the hospital setting as well.

In addition to Exandra, there have been several other CDSSs developed in various therapy areas that have displayed both advantages and limitations. For instance, one CDSS used patient characteristics to identify individuals with T2D and determine the level of risk to their health status [17]. While this CDSS provided risk detection rather than treatment recommendations, it showed a high level of accuracy when validated using four separate case studies, and could be advantageous for preventing critical medical situations in patients with T2D. Additionally, several CDSSs have been developed to provide cardiovascular risk assessment [18, 19]. Other CDSSs were successfully developed using information from specialist recommendations alone [23] or alongside recent literature [24], rather than formalized guidelines. This approach could be more practical when clinical practice guidelines are not readily available in the therapeutic area of interest.

The results of implementing CDSSs into clinical practice have proven to be beneficial in many cases. Several CDSSs were found to increase adherence to clinical practice guidelines and increase the detection of potential errors in implementing recommended treatment [21, 25, 26], while others aimed to save healthcare professionals’ time in diagnosing and treating patients [27, 28]. Some have found that the use of CDSSs can improve healthcare in settings where resources are limited [20], and can reduce healthcare costs [21]. CDSSs allow informed shared decision-making and patient awareness/participation within clinical practice. Despite the reported success of implementing CDSSs in clinical practice, developers of these systems should proceed with some caution. In one particular case, a CDSS developed to detect cervical spine fractures provided poor diagnostic accuracy when tested, emphasizing the need for rigorous evaluation studies prior to deployment of these systems [29]. The present study offers a thorough method for validation that could be used in future development of CDSSs.

Limitations of this study included that a simplified version of the interface was used for the validation, leading to some reviewers not agreeing with certain recommendations of Exandra. However, any confusion caused by the simplified interface was clarified by demonstrating the full version of the user interface to reviewers during Phase 2 of the validation. Additionally, because each chapter of Exandra was validated individually based on physician expertise, a full clinical picture was not presented to each reviewer, as it would be for actual users of Exandra. These discrepancies were discussed and clarified during Phase 2 interviews. Another limitation was that updates to Diabetes Canada’s clinical guidelines for diabetes are released each year, which may have led some reviewers to disagree with Exandra’s recommendations based on their current up-to-date knowledge. In order to remain viable, Exandra should be updated to keep current as new guideline updates are published. Finally, there was a possibility of bias or human error among the reviewers of Exandra. To control for potential bias or error, the secondary outcome of the proportion of PN cases in which the reviewers agreed with the incorrect treatment recommendations helped to verify that bias did not greatly alter the validation of Exandra. It was possible the reviewers may have been more likely to respond “yes” to the cases to avoid spending more time making mandatory comments. Nonetheless, the reviewers were leading experts in their field and were compensated for their time, which likely motivated them to provide thoughtful comments and accurately respond to the Exandra validation. Additionally, there were many cases in which reviewers left optional comments on “yes” responses, suggesting it was unlikely they avoided answering “no” or “not sure” to avoid having to make comments.

## Conclusions

Exandra displayed a high level of accuracy and precision in providing recommendations for managing T2D and its common comorbidities. Exandra is able to offer a certain level of personalization within the framework of its sources. It is important to note, however, that recommendations made by Exandra are suggestive and not authoritative, and actual users are asked through disclaimers to only adopt the suggestions according to their clinical discretion. In addition to the validation study presented here, randomized clinical trials and real-world evidence studies are still needed to evaluate the usability, acceptability, and efficacy of Exandra. The results of this study indicate that Exandra is a promising tool for improving the management of T2D and its comorbidities.

## Supplementary Information

Supplementary Material 1.

## Data Availability

All relevant data are reported in the paper.

## Abbreviations

CDSS: Clinical decision support system
CI: Confidence interval
DPP-4: Dipeptidyl peptidase-4
FP: False positive
GLP-1RA: Glucagon-like peptide-1 receptor agonist
PN: Predicted negative
PP: Predicted positive
SGLT2i: Sodium-glucose cotransporter-2 inhibitor
T2D: Type 2 diabetes
TP: True positive
