# Supplementary material for: Analytical validation of Exandra: a clinical decision support system for promoting guideline-directed therapy of type-2 diabetes in primary care – a collaborative study with experts from Diabetes Canada
Source: BMC Med Inform Decis Mak. 2025 Feb 12;25:74. doi: 10.1186/s12911-025-02881-4 (PMC11816501; doi:10.1186/s12911-025-02881-4)
Supplement: Supplementary file 1 — Supplementary Material 1. [file 12911_2025_2881_MOESM1_ESM.docx]

**SUPPLEMENT**

**Analytical validation of Exandra: a clinical decision support system for promoting guideline-directed therapy of type-2 diabetes in primary care – a collaborative study with experts from Diabetes Canada**

Klaudia Grechuta^1*†^, Pedram Shokouh^2*^, Valentina Bayer^3^, Henrich Kraemer^1^, Jeremy Gilbert^4^, Susie Jin^5^, Ahmad Alhussein ^1^

*Co-first authors

^†^Corresponding author

^1^Boehringer Ingelheim International GmbH, Ingelheim am Rhein, Germany; ^2^Adivus Medical Consultancy Mpv., Aarhus, Denmark; ^3^Boehringer Ingelheim Pharmaceuticals, Inc., Ridgefield, CT, USA; ^4^Sunnybrook Health Sciences Centre, University of Toronto, Toronto, Canada; ^5^Clinical Pharmacist, Certified Diabetes Educator, Ontario, Canada

**Supplementary Methods**

**Supplementary Table S1.** List of references used to build the Exandra engine

| **Chapter** | **Primary vs supporting** | **Reference** |
| --- | --- | --- |
| Chapter 1: Glycemic management | Primary | Diabetes Canada 2018 Clinical Practice Guidelines for the Prevention and Management of Diabetes in Canada: Chapter 13. Pharmacologic Glycemic Management of Type 2 Diabetes in Adults: 2020 Update |
|  |  | Diabetes Canada 2018 Clinical Practice Guidelines for the Prevention and Management of Diabetes in Canada: Chapter 13. Pharmacologic Glycemic Management of Type 2 Diabetes in Adults: 2020 Update — The User’s Guide |
|  | Supporting | Diabetes Canada 2018 Clinical Practice Guidelines for the Prevention and Management of Diabetes in Canada: Chapter 13. Pharmacologic Glycemic Management of Type 2 Diabetes in Adults |
| Chapter 2: Dyslipidemia | Primary | Diabetes Canada 2018 Clinical Practice Guidelines for the Prevention and Management of Diabetes in Canada: Chapter 23. Cardiovascular Protection in People with Diabetes |
|  | Supporting | 2021 Canadian Cardiovascular Society Guidelines for the Management of Dyslipidemia for the Prevention of Cardiovascular Disease in Adults |
|  |  | 2016 Canadian Cardiovascular Society Guidelines for the Management of Dyslipidemia for the Prevention of Cardiovascular Disease in the Adult |
| Chapter 3: Blood pressure control | Primary | Diabetes Canada 2018 Clinical Practice Guidelines for the Prevention and Management of Diabetes in Canada: Chapter 26. Treatment of Hypertension |
|  | Supporting | 2020 Hypertension Canada’s Comprehensive Guidelines for the Prevention, Diagnosis, Risk Assessment, and Treatment of Hypertension in Adults and Children |
| Chapter 4: Anti-platelet therapy | Primary | Diabetes Canada 2018 Clinical Practice Guidelines for the Prevention and Management of Diabetes in Canada: Chapter 23. Cardiovascular Protection in People with Diabetes |
|  | Supporting | 2018 Canadian Cardiovascular Society/Canadian Association of Interventional Cardiology Dyslipidemia Focused Update of the Guidelines for the Use of Antiplatelet Therapy |

**Supplementary Table S2.** Patient parameters used to define clinical scenarios

| **Parameters** | **USML Code** | **Type** |
| --- | --- | --- |
| Age (years) | C0001779 | Integer |
| Sex | C1522384 | Boolean |
| Current HbA1c | C0019018 | Float |
| Target HbA1c | - | Float |
| Metabolic decompensation | C3804961 | Boolean |
| DM duration >15 years | - | Boolean |
| Retinopathy | C0011884 | Boolean |
| Neuropathy | C0011882 | Boolean |
| Atherosclerotic CVD | C0007222 | Boolean |
| Heart Failure | C0018801 | Boolean |
| LVH | C0149721 | Boolean |
| CKD | C0011881 | Boolean |
| eGFR | C4524116 | Integer |
| LDL-C | C0023823 | Float |
| HDL-C | C0023821 | Float |
| Total cholesterol | C0543421 | Float |
| TG (mmol/L) | C0041004 | Float |
| Familial hypercholesterolemia | C0020445 | Boolean |
| Is LDL-C reduced by 50%? | - | Boolean |
| Systolic BP | C0871470 | Float |
| Diastolic BP | C0428883 | Float |
| Smoking | C0037369 | Integer |
| Central obesity | C0311277 | Boolean |
| Current medication | - | Text |

BP, blood pressure; CKD, chronic kidney disease; CVD, cardiovascular disease; DM, diabetes mellitus; eGFR, estimated glomerular filtration rate; HbA1c, glycated hemoglobin; HDL-C, high -density lipoprotein cholesterol; LDL-C, low-density lipoprotein cholesterol; LVH, left ventricular hypertrophy; TG, triglycerides

**Sample size determination**

Since the proportion *p* of cases labeled correctly by Exandra out of the PPs was unknown, it was assumed that the width of the 90% confidence interval around *p* was at most 10%, which corresponded to a margin of error *d* = 5%. In the worst case, *p* = 50% (i.e., half of the PP cases were incorrectly labeled). The required sample size was calculated with the formula below for the binomial distribution; the sample size depends on the population size [1]].

1. Large population

n = z^2 * p*(1-p)/ d^2

p*(1-p) reaches its maximum 0.25 when *p* = 0.5

for a 90% confidence interval, *d* = 0.05 and *z* = 1.645, so the sample size *n* ≥ 271

2. Small population (of size N)

n’ = N * n / (n+N), where *n* is computed with the formula in step 1 above. The Antiplatelet chapter (Chapter 4) had only 160 total cases, so *N* = 160, and with *n* = 271, the resulting adjusted sample size is n’ = 101.

**Supplementary References**

1. Vallejo A, Muniesa A, Ferreira C, de Blas I. New method to estimate the sample size for calculation of a proportion assuming binomial distribution. Res Vet Sci. 2013;95(2):405-9.
